# Supplementary material for: Sequencing and Chromosome-Scale Assembly of Plant Genomes, Brassica rapa as a Use Case
Source: Biology (Basel). 2021 Jul 30;10(8):732. doi: 10.3390/biology10080732 (PMC8389630; doi:10.3390/biology10080732)

Table S1. Metrics of Nanopore datasets

Readset	All reads	Longest reads	Filtlong reads
Cumulative size	93,615,101,387	15,000,004,992	15,000,009,495
Coverage	186x	30x	30x
Number of reads	13,094,187	214,687	316,605
N50	26,978	68,127	50,558
Number of reads > 50kb	296,164	214,687	117,159
coverage > 50kb	38x	30x	15x

Table S2. Metrics of raw Smartdenovo Nanopore assemblies. Statistics were generated on contigs of more than 30kb in size.

Readset	All reads	Longest reads	Filtlong reads
Cumulative size	413,393,283	354,455,409	320,273,956
Number of contigs	596	323	429
N50 (L50)	10,591,557 (14)	4,616,132 (19)	2,264,044 (35)
N90 (L90)	246,123 (185)	326,576 (146)	263,776 (203)
auN	9,674,642	6,312,816	3,679,656
Max. size	27,174,673	18,229,467	17,762,612
Complete buscos (%)	1,576 (97.6%)	1,436 (88.9%)	1,557 (96.5%)
Merqury score	25.1333	20.3158	23.8738

Table S3. Metrics of raw Flye Nanopore assemblies. Statistics were generated on contigs of more than 30kb in size.

Readset	All reads	Longest reads	Filtlong reads
Cumulative size	308,405,611	340,681,490	320,203,564
Number of contigs	195	266	382
N50 (L50)	8,877,964 (12)	13,456,414 (10)	6,180,078 (13)
N90 (L90)	974,980 (48)	497,751 (58)	310,436 (106)
auN	9,118,954	12,335,383	8,103,042
Max. size	21,466,788	27,098,069	22,540,294
Complete buscos (%)	1,585 (98.2%)	1,584 (98.1%)	1,594 (98.8%)
Merqury score	28.7338	27.4808	28.0033

Table S4. Metrics of raw Wtdbg2 Nanopore assemblies. Statistics were generated on contigs of more than 30kb in size.

Readset	All reads	Longest reads	Filtlong reads
Cumultive size	728,902,491	541,375,314	381,524,645
Number of contigs	9,550	5,095	3,213
N50 (L50)	87,609 (2,265)	142,868 (574)	206,244 (357)
N90 (L90)	38,374 (7,405)	42,678 (3,598)	44,750 (2,179)
auN	144,875	654,911	450,988
Max. size	1,853,134	6,900,900	3,496,279
Complete buscos (%)	1,455 (90.1%)	1,365 (84.6%)	1,402 (86.9%)
Merqury score	17.4181	15.9445	18.4839

Table S5. Metrics of the raw Necat Nanopore assembly. Statistics were generated on contigs of more than 30kb in size.

Readset	All reads
Cumulative size	442,919,932
Number of contigs	299
N50 (L50)	10,445,217 (12)
N90 (L90)	856,761 (58)
auN	14,175,039
Max. size	45,040,585
Complete buscos (%)	1,567 (97.1%)
Merqury score	27.513

Table S6. Metrics of the Necat assembly after each polishing step. Statistics were generated on contigs of more than 30kb in size.

	**Raw**	**Racon**	**Medaka**	**Hapo-G x2**
Cumulative size	442,919,932	443,505,353	443,619,512	443,649,441
Number of contigs	299	299	299	299
N50 (L50)	10,445,217 (12)	10,458,156 (12)	10,461,728 (12)	10,461,875 (12)
N90 (L90)	856,761 (58)	857,014 (58)	850,928 (58)	857,267 (58)
auN	14,175,039	14,197,548	14,204,585	14,202,687
Max. size	45,040,585	45,103,281	45,117,601	45,115,632
Complete buscos (%)	1,567 (97.1%)	1,592 (98.6%)	1,602 (99.3%)	1,604 (99.4%)
Merqury score	27.513	29.8573	31.0464	36.4423

Table S7. Hybrid scaffolding results.

	Nanopore contigs	Hybrid scaffolds	Contigs left	Assembly after Hybrid scaffoldin g	Scaffolds after BisCoT treatment	Contigs after BisCoT treatment	Scaffolds after polishing with Hapo-G
Number	299	53	208	261	236	296	236
N50 (L50)	10,461,875 (12)	16,816,852 (8)	154,636 (53)	16,816,852 (8)	17,017,530 (8)	10,256,302 (14	17,017,634 (8)
N90 (L90)	857,267 (58)	4,034,000 (45)	67,775 (152)	1,954,922 (32)	3,409,196 (30)	981,751 (59)	3,409,175 (30)
Min size	30,349	118,769	30,349	30,349	30,347	30,347	30,348
Max size	45,115,632	44,052,815	978,115	44,052,815	44,068,965	27,443,240	44,069,534
Cumulati ve size	443,649,441	420,416,306	26,002,488	446,418,794	443,948,706	441,033,740	443,951,349
%N	0%	0.7%	0%	0.66%	0.66%	0%	0.66%

Table S8. Metrics of the Pore-C raw PromethION run with (left column) or without (right column) reads of more than 100kb in size.

Readset	All reads	Reads < 100kb
Cumulative size	15,551,590,510	15,502,009,110
Number of reads	5,894,741	5,894,524
N50	3,990	3,990
Number of reads > 100kb	217	0
Cumulative size > 100kb	49,581,400	0

Figure S1. Dotplots of polished Necat contigs aligned to *Brassica rapa* Chiifu (left) or *Brassica napus* Darmor-BZH A genome (right).

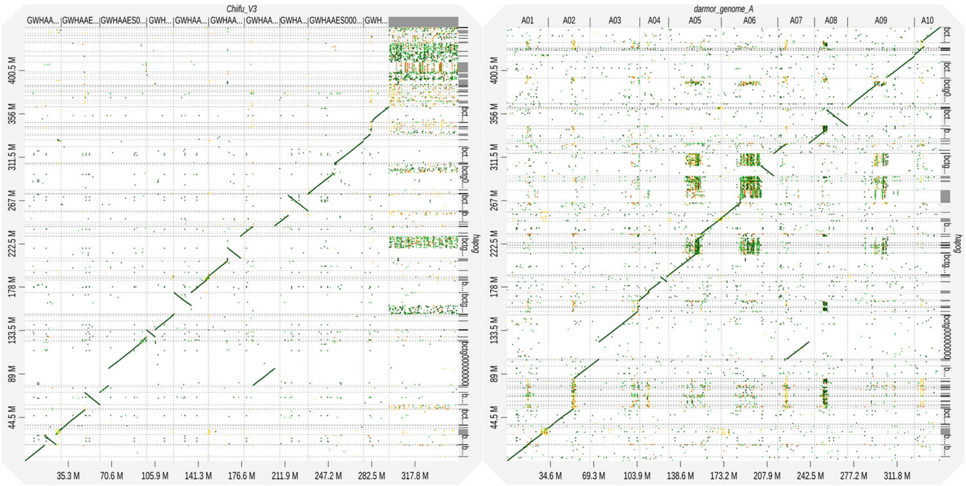

Figure S2. Dotplots of Bionano scaffolds aligned to *Brassica rapa* Chiifu (left) or *Brassica napus* Darmor-BZH A genome (right).

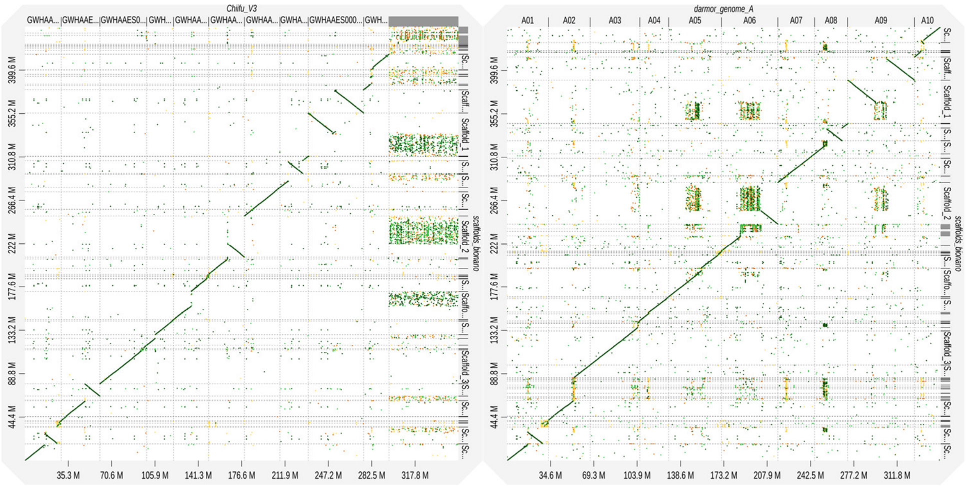

Figure S3. Dotplots of Omni-C scaffolds aligned to *Brassica rapa* Chiifu (left) or *Brassica napus* Darmor-BZH A genome (right).

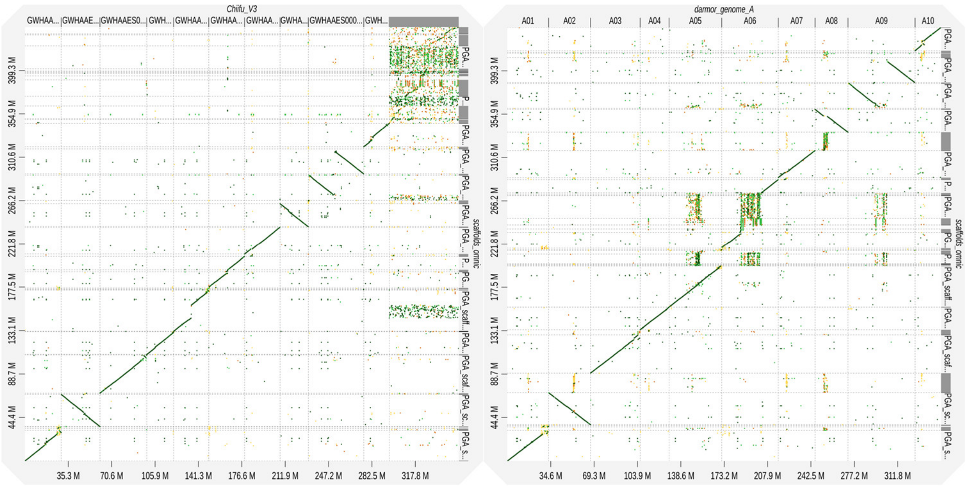

Figure S4. Dotplots of Pore-C scaffolds aligned to *Brassica rapa* Chiifu (left) or *Brassica napus* Darmor-BZH A genome (right).

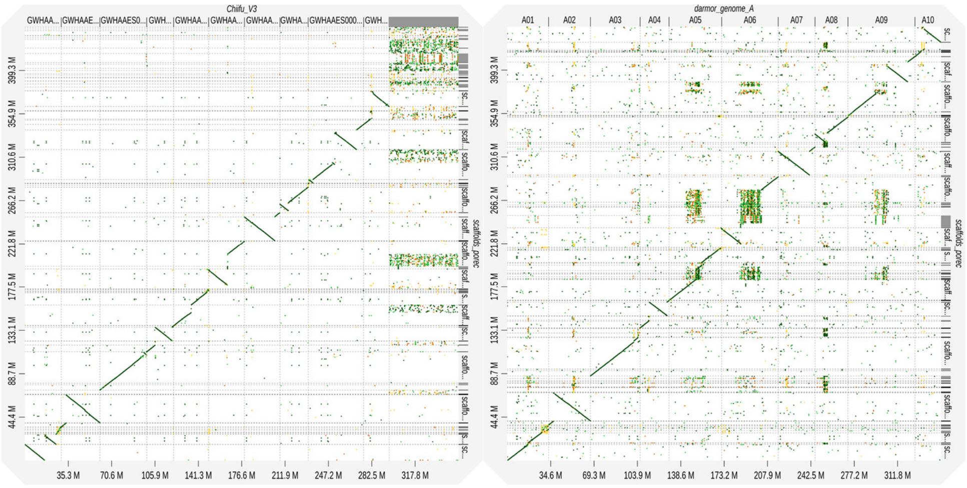

Figure S5. Dotplots of Bionano + Omni-C scaffolds aligned to *Brassica rapa* Chiifu (left) or *Brassica napus* Darmor-BZH A genome (right).

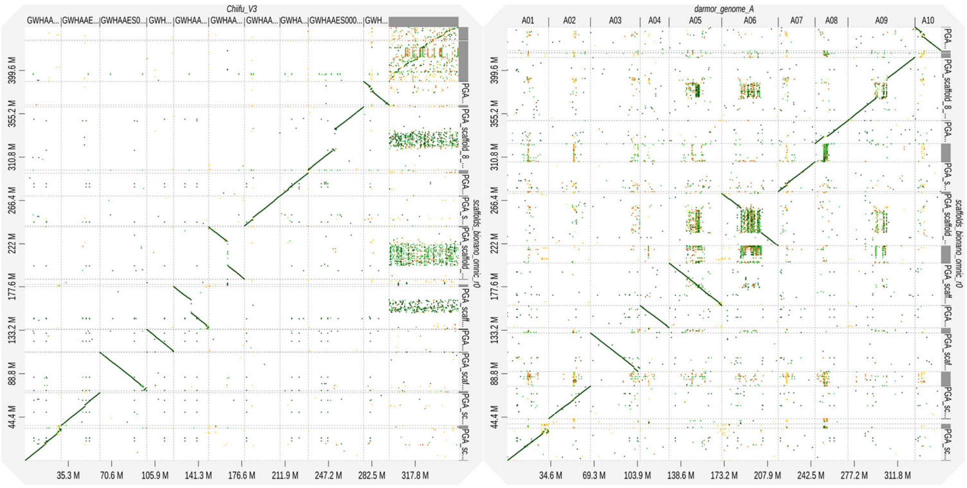

Figure S6. Dotplots of Bionano + Pore-C scaffolds aligned to *Brassica rapa* Chiifu (left) or *Brassica napus* Darmor-BZH A genome (right).

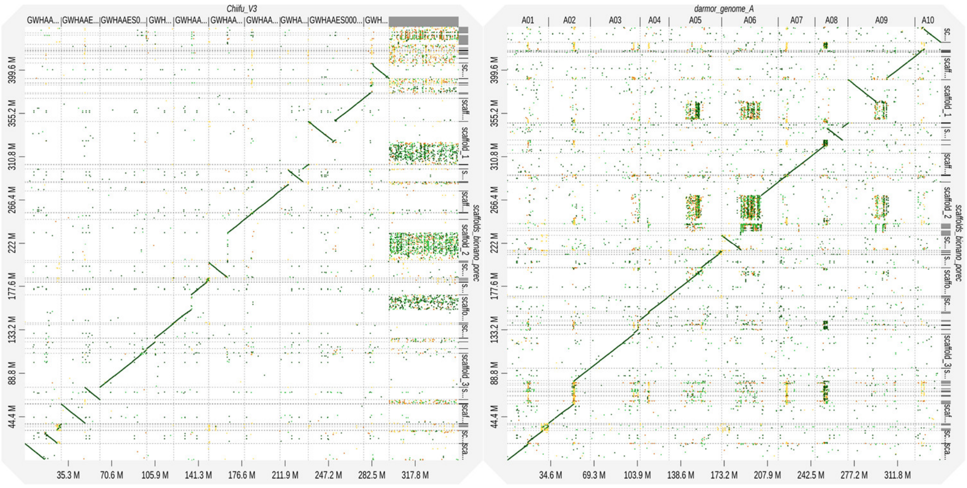

Figure S7. Dotplots of Omni-C + Bionano scaffolds aligned to *Brassica rapa* Chiifu (left) or *Brassica napus* Darmor-BZH A genome (right).

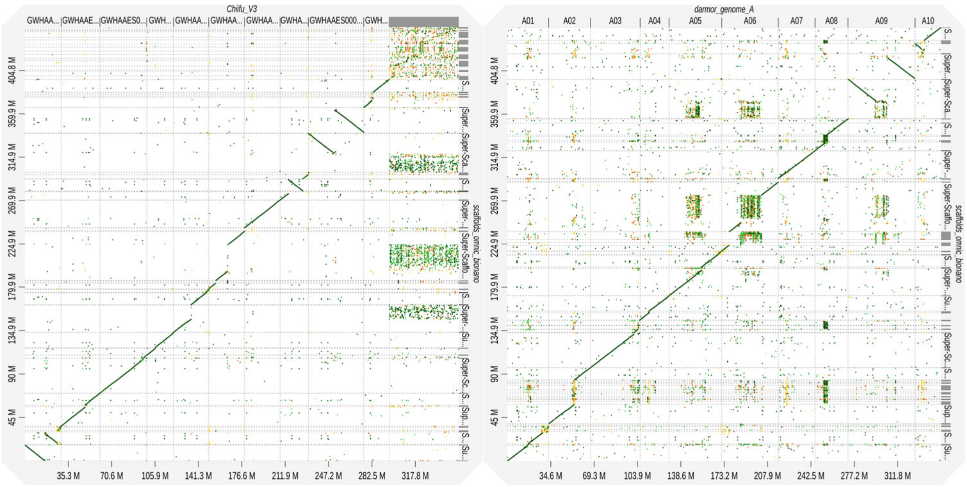

Figure S8. Dotplots of Pore-C + Bionano scaffolds aligned to *Brassica rapa* Chiifu (left) or *Brassica napus* Darmor-BZH A genome (right).

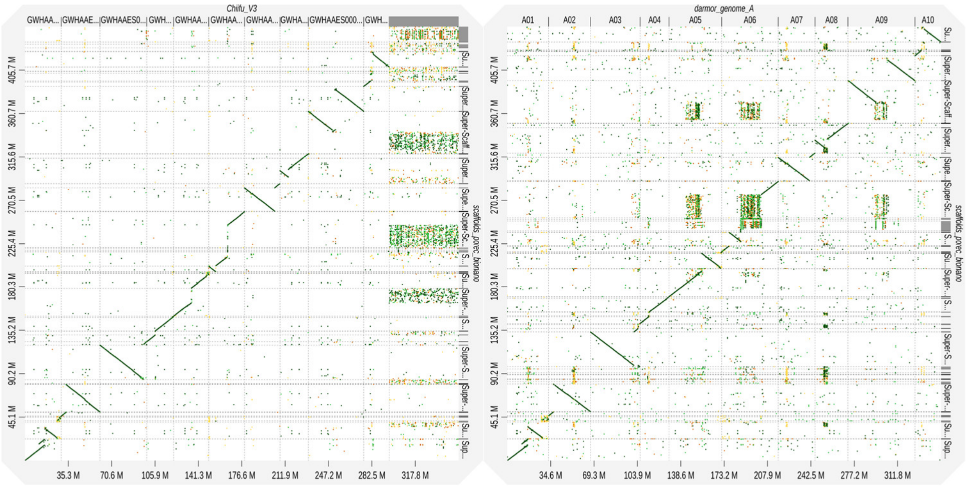

Figure S9. Contact Map generated with the Omni-C library (mapping on the nanopore contigs). The left panel shows the contact before review. The right panel shows the contact map after review.

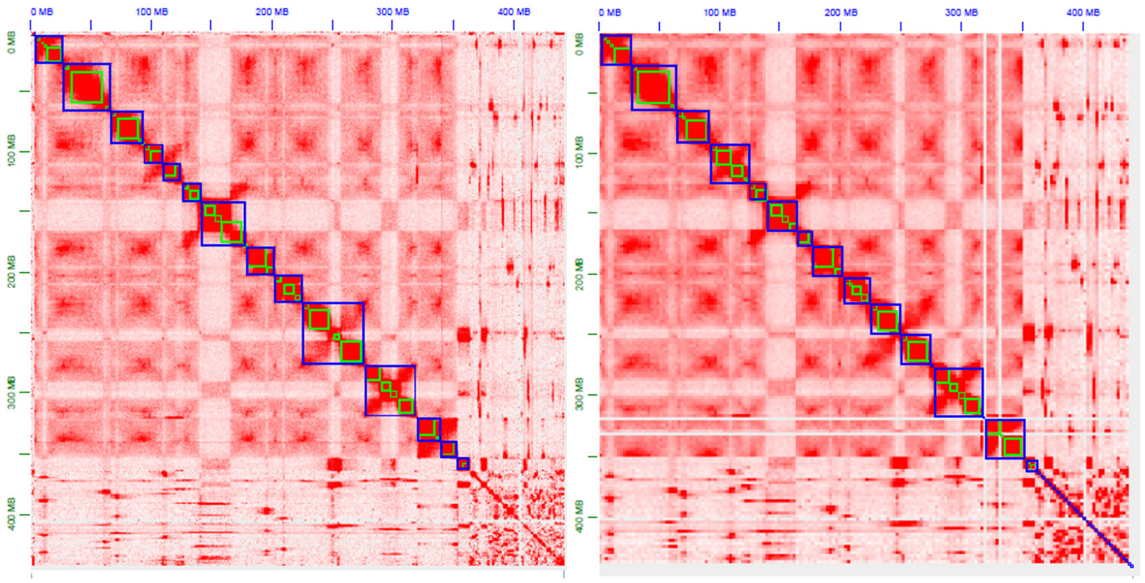

Figure S10. Contact Map generated with the Omni-C library (mapping on the bionano scaffolds). The left panel shows the contact before review. The right panel shows the contact map after review.

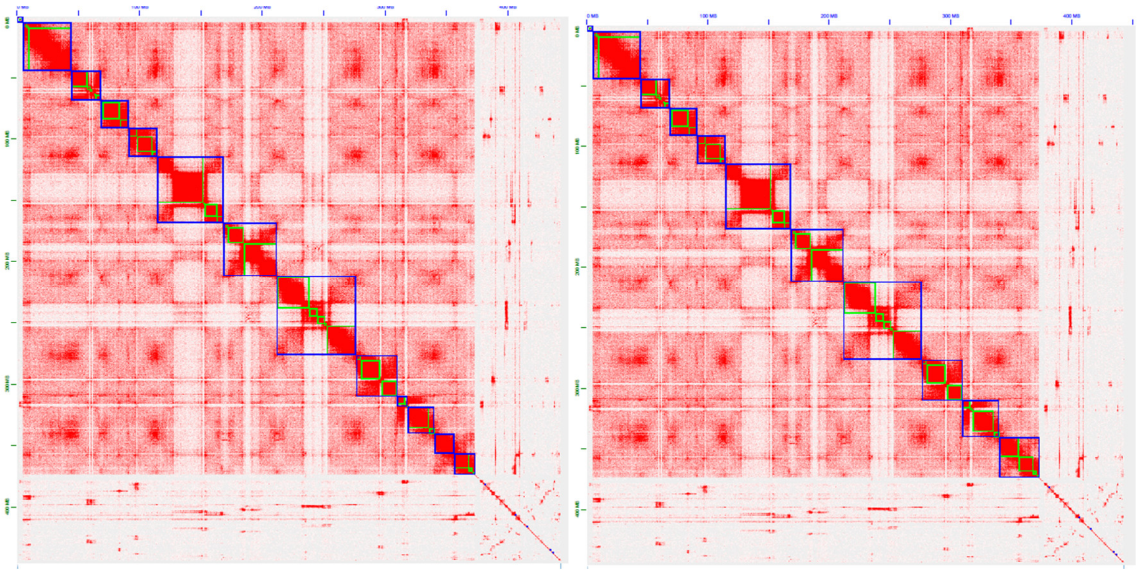

Figure S11. Conflict example detected by the optical maps on the Omni-C scaffolds. The two first lines represent the DLE-1 maps. The third line represents one Omni-C scaffold. The two last lines represent the BspQI maps. The vertical lines show the correspondence between the labels of the scaffold and the labels of the maps. In the middle, we can see a region without any correspondence which leads to a break of the scaffold.

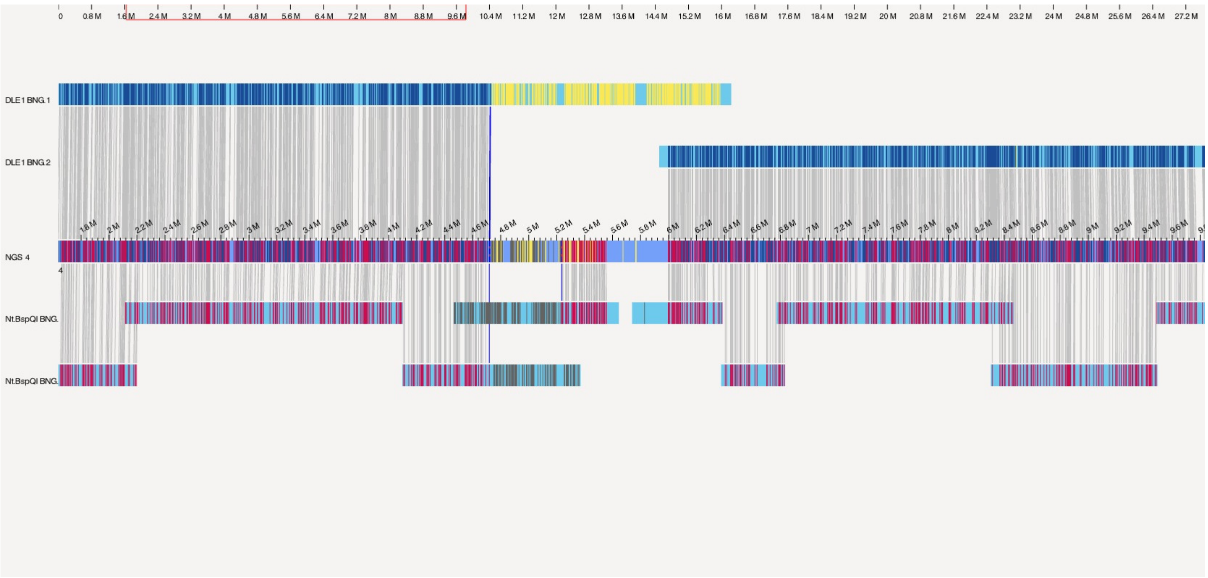

Figure S12. Conflict example detected by the optical maps on the Pore-C scaffolds. The three first lines represent the DLE-1 maps. The fourth line represents one Pore-C scaffold. The two last lines represent the BspQI maps. The vertical lines show the correspondence between the labels of the scaffold and the labels of the maps. We can see regions without any correspondence (the yellow portion of the DLE-1 maps and the grey portions of the BspQI maps) which leads to several breaks of the scaffold.

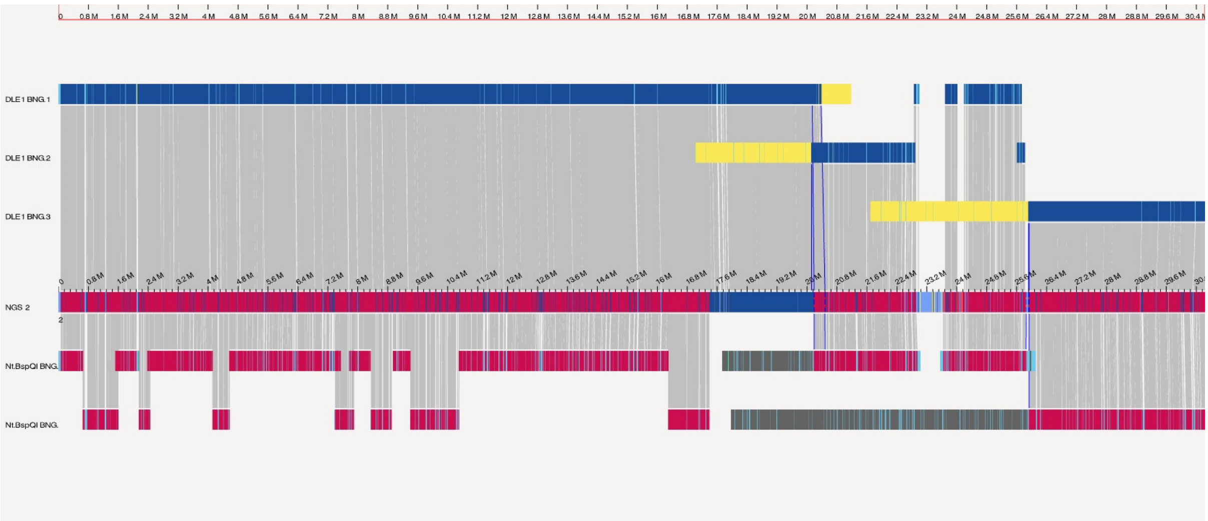

Supplement: Supplementary file 1 [file biology-10-00732-s001.zip › biology-1314133-supplementary.pdf]
